# Analysis of COVID-19 Vaccine Type and Adverse Effects Following Vaccination

**DOI:** 10.1001/jamanetworkopen.2021.40364

**Published:** 2021-12-22

**Authors:** Alexis L. Beatty, Noah D. Peyser, Xochitl E. Butcher, Jennifer M. Cocohoba, Feng Lin, Jeffrey E. Olgin, Mark J. Pletcher, Gregory M. Marcus

**Affiliations:** 1 Department of Epidemiology and Biostatistics, University of California, San Francisco; 2 Division of Cardiology, Department of Medicine, University of California, San Francisco; 3 Department of Clinical Pharmacy, University of California San Francisco School of Pharmacy

## Abstract

**Importance:**

Little is known about the factors associated with COVID-19 vaccine adverse effects in a real-world population.

**Objective:**

To evaluate factors potentially associated with participant-reported adverse effects after COVID-19 vaccination.

**Design, Setting, and Participants:**

The COVID-19 Citizen Science Study, an online cohort study, includes adults aged 18 years and older with a smartphone or internet access. Participants complete daily, weekly, and monthly surveys on health and COVID-19–related events. This analysis includes participants who provided consent between March 26, 2020, and May 19, 2021, and received at least 1 COVID-19 vaccine dose.

**Exposures:**

Participant-reported COVID-19 vaccination.

**Main Outcomes and Measures:**

Participant-reported adverse effects and adverse effect severity. Candidate factors in multivariable logistic regression models included age, sex, race, ethnicity, subjective social status, prior COVID-19 infection, medical conditions, substance use, vaccine dose, and vaccine brand.

**Results:**

The 19 586 participants had a median (IQR) age of 54 (38-66) years, and 13 420 (68.8%) were women. Allergic reaction or anaphylaxis was reported in 26 of 8680 participants (0.3%) after 1 dose of the BNT162b2 (Pfizer/BioNTech) or mRNA-1273 (Moderna) vaccine, 27 of 11 141 (0.2%) after 2 doses of the BNT162b2 or mRNA-1273 vaccine or 1 dose of the JNJ-78436735 (Johnson & Johnson) vaccine. The strongest factors associated with adverse effects were vaccine dose (2 doses of BNT162b2 or mRNA-1273 or 1 dose of JNJ-78436735 vs 1 dose of BNT162b2 or mRNA-1273; odds ratio [OR], 3.10; 95% CI, 2.89-3.34; *P* < .001), vaccine brand (mRNA-1273 vs BNT162b2, OR, 2.00; 95% CI, 1.86-2.15; *P* < .001; JNJ-78436735 vs BNT162b2: OR, 0.64; 95% CI, 0.52-0.79; *P* < .001), age (per 10 years: OR, 0.74; 95% CI, 0.72-0.76; *P* < .001), female sex (OR, 1.65; 95% CI, 1.53-1.78; *P* < .001), and having had COVID-19 before vaccination (OR, 2.17; 95% CI, 1.77-2.66; *P* < .001).

**Conclusions and Relevance:**

In this real-world cohort, serious COVID-19 vaccine adverse effects were rare and comparisons across brands could be made, revealing that full vaccination dose, vaccine brand, younger age, female sex, and having had COVID-19 before vaccination were associated with greater odds of adverse effects. Large digital cohort studies may provide a mechanism for independent postmarket surveillance of drugs and devices.

## Key Points

**Question:**

What factors are associated with adverse effects after COVID-19 vaccination?

**Findings:**

In an online cohort study including 19 586 adults who received a COVID-19 vaccination, the factors most strongly associated with adverse effects were full vaccination dose, brand of vaccine, younger age, female sex, and having had COVID-19 before vaccination. Allergic reaction or anaphylaxis was reported in 0.3% of participants after partial vaccination and 0.2% of participants after full vaccination.

**Meaning:**

These findings suggest that some individuals experience more adverse effects after COVID-19 vaccination, but serious adverse effects are rare.

## Teaser

This cohort study evaluates factors potentially associated with participant-reported adverse effects after COVID-19 vaccination.

## Introduction

In December 2020, the first COVID-19 vaccines received emergency use authorization in the United States.^1,2^ Billions of doses of vaccine have been administered worldwide.^3^ However, some individuals have concerns about receiving COVID-19 vaccination related to vaccine safety and adverse effects.^4^

In randomized clinical trials of COVID-19 vaccines, reported adverse effects included injection site events (eg, pain, redness, swelling) and systemic effects (eg, fatigue, headache, muscle or joint pain), with rare serious adverse events.^5,6,7,8^ Most adverse effects were mild, but studies reported approximately 50% to 90% of participants experiencing some adverse effects.^5,6,8^ Although data have begun to emerge on adverse effects reported through government-sponsored reporting systems,^9,10,11,12,13^ there is little real-world, patient-reported data on adverse effects after receiving COVID-19 vaccination and in whom adverse effects may be more common.

The objectives of this study were to describe adverse effects and identify factors associated with adverse effects after COVID-19 vaccination in participants in an online cohort study. In addition, the study sought to identify factors associated with more severe adverse effects. These results may help the public gain a greater understanding of the real-world experience of adverse effects after COVID-19 vaccination.

## Methods

### Design, Setting, and Participants

The COVID-19 Citizen Science (CCS) study is an online cohort study that began enrolling participants on March 26, 2020.^14^ CCS is hosted on the Eureka Research Platform (University of California, San Francisco), a digital platform for clinical research studies including a mobile application (app) and web-based software. Participants are recruited to the study through email invitations to participants in other Eureka Research Platform studies, press releases, word-of-mouth, and recruitment through partner organizations. Participants must be 18 years or older, register for a Eureka Research Platform account, have an iOS or Android smartphone with a cellular phone number (or enroll in the web-based study launched January 21, 2021), agree to participate in English, and be able to provide consent to participate in the study. After providing electronic consent, participants complete baseline, daily, weekly, and monthly surveys. CCS methods have been previously described.^14,15,16,17^ For this analysis, we included data collected between March 26, 2020, and May 19, 2021. The study was reviewed and approved by the University of California, San Francisco, institutional review board. Results are reported in accordance with Strengthening the Reporting of Observational Studies in Epidemiology (STROBE) reporting guideline.^18^

### COVID-19 Vaccination

On January 14, 2021, participants began receiving baseline and monthly surveys asking, “Have you ever received a COVID-19 (SARS-CoV-2) vaccine?” Follow-up questions immediately after a participant reported receiving a vaccine asked participants how many doses they received, the dates of vaccine doses, vaccine brand, and where they received the vaccine. Participants received a monthly follow-up survey asking about receiving additional doses of vaccine. Monthly surveys were chosen to limit participant survey burden and because the administration schedules for vaccines with multiple doses separated doses by 3 to 4 weeks. Partial vaccination was defined as receiving 1 dose of any vaccine other than JNJ-78436735 (Johnson & Johnson). Full vaccination was defined as receiving 1 dose of JNJ-78436735 or 2 doses of any other vaccine. At the time these data were collected, third or booster doses were not yet recommended, so data on additional doses were not collected. Final analyses included only participants who reported receiving BNT162b2 (Pfizer/BioNTech), mRNA-1273 (Moderna), or JNJ-78436735 vaccines because of small numbers of receipt of other vaccines in this study.

### Outcomes

After reporting vaccination, participants were asked to report vaccine adverse effects, with response options including fever, chills, fatigue, sore/scratchy throat, muscle pain, joint pain, headache, other pain, redness/swelling at the injection site, rash other than at the injection site, allergic reaction/anaphylaxis, other, and none of the above. These response options were chosen because these adverse effects had been reported in vaccine clinical trials. Participants could provide free-text responses to the option of other. Following branching logic, participants reporting adverse effects were also asked the duration of adverse effects and self-rated adverse effect severity (very mild, mild, moderate, severe, and very severe). If participants reported receiving 2 doses of vaccine on the same survey, they were not asked to report adverse effects by dose separately.

### Other Variables

At baseline, participants reported characteristics, including age, sex, gender, race (American Indian or Alaska Native, Asian, Black or African American, Native Hawaiian or Pacific Islander, White, and other or do not know), ethnicity, zip code, Macarthur subjective social status (rated 0-10, with 10 being the highest),^19^ highest educational attainment, primary employment, working from home, regular contact with people 65 years and older, receipt of influenza shot in the past year, medical conditions, and current tobacco and marijuana use. Rural and urban status was determined using 2010 zip code and rural-urban commuting area codes from the US Department of Agriculture.^20^ Median income by zip code was determined using American Community Survey 5-year estimates from 2019.^21^ Participants provide current medication lists, which are indexed to RxNorm.

Participants reported results of COVID-19 testing and date of testing at baseline and on weekly surveys. In this study, we defined COVID-19 as a participant-reported positive test for active infection (polymerase chain reaction or antigen testing).

### Statistical Analysis

Descriptive statistics including mean, SD, median, and IQR are used to describe baseline characteristics and questionnaire responses. For baseline characteristics and survey responses, differences in characteristics were examined with a *t* test or Kruskal-Wallis test for continuous variables and χ^2^ test for categorical variables. A multivariable logistic regression model was constructed to identify factors associated with any adverse effects (vs no adverse effects). Given that vaccination and adverse effects were queried monthly, participants could report 2 doses of vaccine in 1 survey. When participants reported 2 doses of vaccine in a monthly survey, the reported adverse effects were associated with the second vaccine dose. Candidate factors in the multivariable models (with all factors entered into the model simultaneously) included age (as a continuous variable, per 10 years), sex assigned at birth (female or all others), race (Asian, Black or African American, multiracial, White, or other), Hispanic ethnicity, subjective social status, medical conditions (hypertension, diabetes, myocardial infarction, coronary heart disease, heart failure, stroke or transient ischemic attack, atrial fibrillation, obstructive sleep apnea, chronic obstructive pulmonary disease, asthma, immunodeficiency, HIV, anemia, and pregnancy), influenza shot in the past year, current tobacco use, current marijuana use, COVID-19 prior to vaccination, vaccine dose, and vaccine brand (BNT162b2, mRNA-1273, or JNJ-78436735). A separate multivariable logistic regression model was constructed to identify factors associated with severe or very severe adverse effects (vs no, very mild, mild, or moderate adverse effects) using the same candidate factors. We conducted an exploratory analysis of adverse effects in participants with asthma with and without use of inhaled corticosteroids. Statistical significance was considered to be *P* < .05, and all tests were 2-tailed. All analyses were conducted with SAS version 9.4 (SAS Institute).

## Results

As of May 19, 2021, 19 586 participants reported receiving at least 1 dose of vaccine, with a median (IQR) age of 54 (38-66) years and 13 492 (68.8%) female participants (Table 1). In the overall cohort, 65 921 participants had enrolled, and 46 204 (70%) remained active in the study (ie, completed at least 1 survey in 2021) (eTable 1 in the Supplement). Among 12 215 participants who reported where they were vaccinated (62.3%), the most common sites were at a doctor’s office, clinic, or hospital (5307 [43.4%]), workplace (1834 [15.0%]), public health department (1685 [13.8%]), pharmacy (1217 [10.0%]), and health fair or other public event (1025 [8.4%]).

**Table 1.** Baseline Characteristics of COVID-19 Citizen Science Study Participants Reporting COVID-19 Vaccination

Characteristic	Participants, No. (%)		
	≥1 Vaccination dose (N = 19 586)	Vaccinated	
		Partially (n = 8682)^a^	Fully (n = 11 141)^b^
US resident	19 488 (99.5)	8621 (99.3)	11 103 (99.7)
Age, median (IQR), y	54.0 (38.0-66.0)	49.0 (35.0-61.0)	57.0 (40.0-69.0)
Female sex assigned at birth	13 420 (68.8)	6070 (70.2)	7520 (67.9)
Gender identity			
Male	6024 (30.9)	2558 (29.6)	3528 (31.9)
Female	13 281 (68.1)	5984 (69.2)	7464 (67.4)
Transgender			
Woman	12 (0.1)	5 (0.1)	7 (0.1)
Man	34 (0.2)	24 (0.3)	10 (0.1)
Genderqueer	110 (0.6)	57 (0.7)	57 (0.5)
Other	60 (0.3)	38 (0.4)	24 (0.2)
Race			
American Indian or Alaska Native	286 (1.5)	155 (1.8)	135 (1.2)
Asian	1506 (7.8)	678 (7.9)	844 (7.7)
Black or African American	443 (2.3)	204 (2.4)	246 (2.2)
Native Hawaiian or Pacific Islander	87 (0.4)	34 (0.4)	53 (0.5)
White	17 294 (89.4)	7665 (89.3)	9840 (89.5)
Other or do not know	617 (3.2)	309 (3.6)	319 (2.9)
Hispanic ethnicity	1476 (7.6)	731 (8.5)	767 (6.9)
Rural zip code	1427 (7.3)	625 (7.3)	814 (7.4)
Lives in zip code in lowest quintile for median household income	653 (3.4)	331 (3.9)	331 (3.0)
Subjective social status, mean (SD)^c^	7.0 (1.6)	6.9 (1.6)	7.1 (1.6)
Highest educational level			
No high school degree	40 (0.2)	29 (0.3)	11 (0.1)
High school graduate (or equivalent)	478 (2.5)	239 (2.8)	245 (2.2)
College degree (including associate’s)	9478 (48.6)	4451 (51.5)	5130 (46.3)
Graduate degree	9304 (47.7)	3857 (44.6)	5571 (50.3)
Other	188 (1.0)	73 (0.8)	116 (1.0)
Primary employment			
Health care	5272 (26.9)	1880 (21.7)	3459 (31.1)
Education	2382 (12.2)	1078 (12.4)	1350 (12.1)
Retail	257 (1.3)	144 (1.7)	114 (1.0)
Transportation	190 (1.0)	109 (1.3)	83 (0.7)
Arts, entertainment, and recreation	427 (2.2)	223 (2.6)	209 (1.9)
Hospitality and food services	296 (1.5)	154 (1.8)	148 (1.3)
Finance and insurance	781 (4.0)	434 (5.0)	350 (3.1)
Scientific and technical services	1372 (7.0)	761 (8.8)	629 (5.6)
Utilities	102 (0.5)	63 (0.7)	44 (0.4)
Construction	213 (1.1)	125 (1.4)	91 (0.8)
Manufacturing	328 (1.7)	190 (2.2)	141 (1.3)
Other	7964 (40.7)	3517 (40.5)	4525 (40.6)
Working from home			
100%	1823 (47.0)	1072 (51.8)	773 (41.9)
50%-99%	683 (17.6)	368 (17.8)	324 (17.6)
<50%	514 (13.3)	252 (12.2)	265 (14.4)
None	858 (22.1)	379 (18.3)	483 (26.2)
Regular contact with person aged ≥65 y	9008 (46.0)	3545 (40.8)	5566 (50.0)
Influenza shot in the past year	17 388 (88.8)	7495 (86.3)	10 113 (90.8)
BMI, mean (SD)	27.6 (6.7)	27.9 (7.0)	27.4 (6.5)
Medical condition			
Hypertension	5495 (28.1)	2141 (24.7)	3408 (30.6)
Diabetes	1214 (6.2)	495 (5.7)	733 (6.6)
Coronary artery disease	841 (4.3)	283 (3.3)	562 (5.0)
Myocardial infarction	340 (1.7)	137 (1.6)	203 (1.8)
Congestive heart failure	229 (1.2)	94 (1.1)	137 (1.2)
Stroke or TIA	427 (2.2)	157 (1.8)	274 (2.5)
Atrial fibrillation	946 (4.8)	333 (3.8)	626 (5.6)
Sleep apnea	2447 (12.5)	1006 (11.6)	1463 (13.1)
COPD	472 (2.4)	178 (2.1)	296 (2.7)
Asthma	1939 (9.9)	871 (10.0)	1092 (9.8)
Immunodeficiency	659 (3.4)	299 (3.4)	371 (3.3)
HIV	170 (0.9)	74 (0.9)	98 (0.9)
Anemia	2070 (10.6)	996 (11.5)	1101 (9.9)
Pregnant (at baseline)	236 (1.2)	150 (1.7)	91 (0.8)
Current smoking	691 (3.5)	318 (3.7)	381 (3.4)
Current marijuana use	1677 (8.7)	815 (9.5)	893 (8.1)
COVID-19 (at baseline or incident before date of vaccine)	887 (4.5)	468 (5.4)	432 (3.9)

Abbreviations: BMI, body mass index (calculated as weight in kilograms divided by height in meters squared); COPD, chronic obstructive pulmonary disease; TIA, transient ischemic attack.

^a^ Partially vaccinated is defined as 1 dose of BNT162b2 or mRNA-1273.

^b^ Fully vaccinated is defined as 1 dose of JNJ-78436735 or 2 doses of BNT162b2 or mRNA-1273.

^c^ Subjective social status is rated 1 to 10, with 10 representing the highest perceived social status.

**Figure 1.**
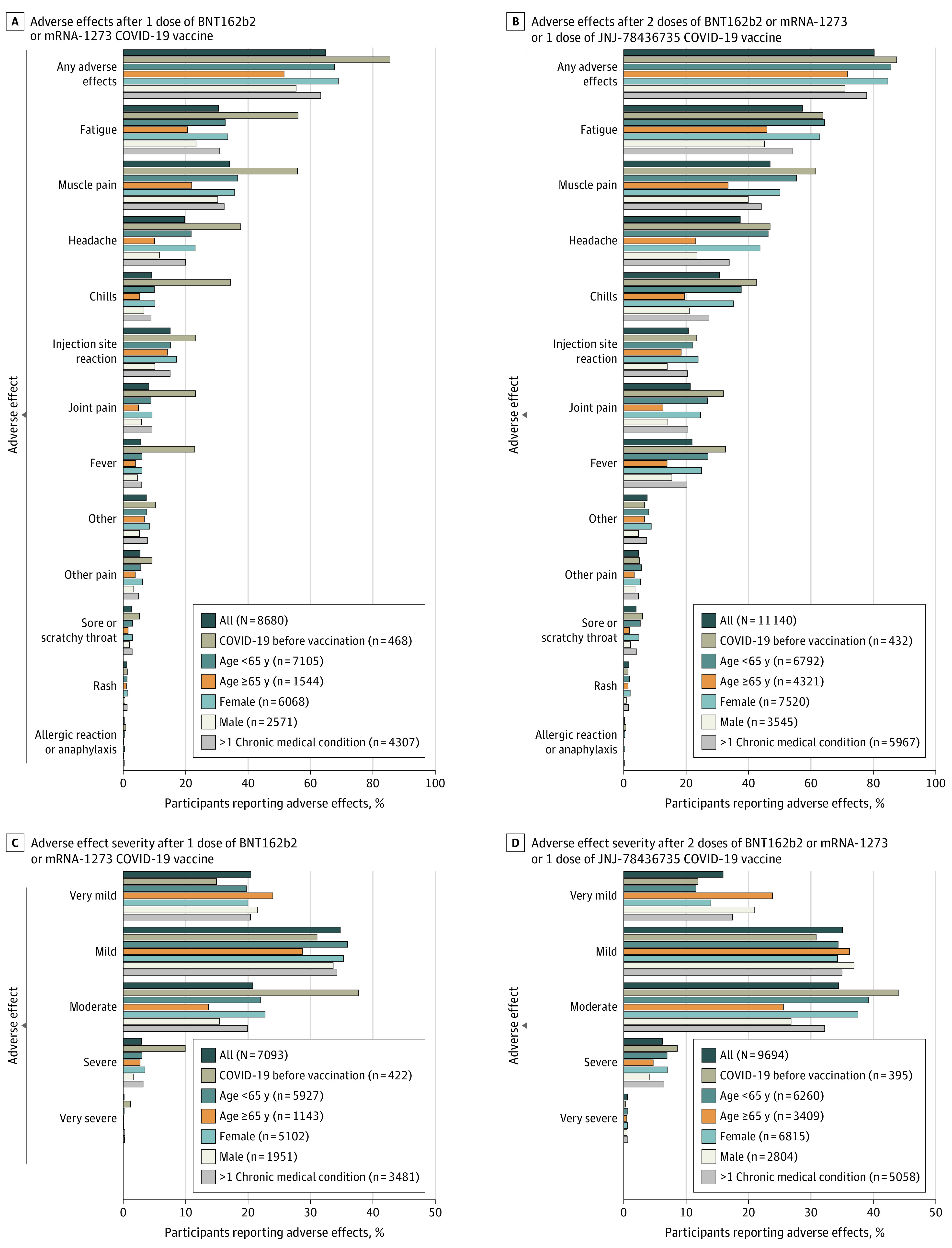
Adverse Effects by Participant Characteristics Participants could report more than 1 adverse effect. Denominators include all participants who provided an answer to the question.

**Figure 2.**
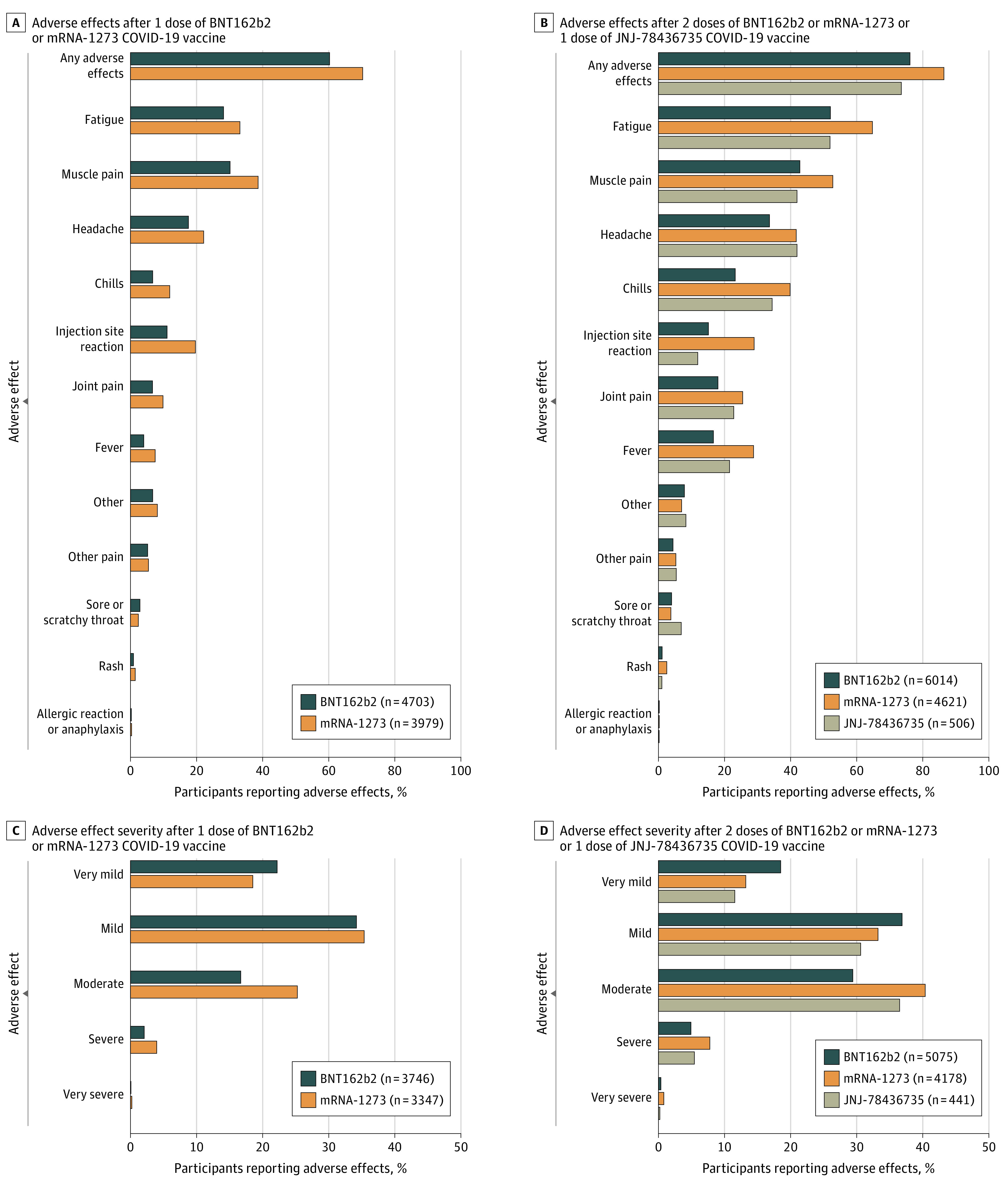
Adverse Effects by Vaccine Brand Participants could report more than 1 adverse effect. Denominators include all participants who provided an answer to the question.

After 1 dose of BNT162b2 or mRNA-1273, 8680 or 8682 participants completed the adverse effects survey and 5629 of 8682 (64.9%) reported adverse effects. After 2 doses of BNT162b2 or mRNA-1273 or 1 dose of JNJ-78436735, 11 140 of 11 141 participants completed the adverse effects survey and 8947 (80.3%) reported adverse effects. The most common vaccine adverse effects were fatigue, muscle pain, headache, chills, redness/swelling at the injection site, joint pain, and fever (Figure 1 and Figure 2). Allergic reaction or anaphylaxis was reported in 26 of 8680 participants (0.3%) after 1 dose of BNT162b2 or mRNA-1273 and 27 of 11 140 participants (0.2%) after 2 doses of BNT162b2 or mRNA-1273 or 1 dose of JNJ-78436735. Other write-in adverse effects included nausea, vomiting, diarrhea, dizziness, brain fog, swollen lymph nodes, and pain/soreness at injection site (eFigure in the Supplement). Two participants reported thrombocytopenia.

In a multivariable logistic regression model examining the association of multiple factors entered into the model simultaneously (age, sex, race, Hispanic ethnicity, subjective social status, medical conditions, influenza shot in the past year, current tobacco use, current marijuana use, COVID-19 prior to vaccination, vaccine dose, and vaccine brand), the factor most strongly associated with the outcome of any adverse effects (compared with no adverse effects) was vaccine dose (2 doses of BNT162b2 or mRNA-1273 or 1 dose of JNJ-78436735 vs 1 dose of BNT162b2 or mRNA-1273: odds ratio [OR], 3.10; 95% CI, 2.89 to 3.34; *P* < .001) (Table 2). Older age (per 10 years: OR, 0.74; 95% CI, 0.72-0.76; *P* < .001), receipt of JNJ-78436735 vaccine (vs BNT162b2), Black or African American race, higher subjective social status, asthma, and anemia were associated with lower odds of reporting adverse effects. Receipt of mRNA-1273 vaccine (vs BNT162b2), female sex (OR, 1.65; 95% CI, 1.53-1.78; *P* < .001), prior COVID-19 (OR, 2.17; 95% CI, 1.77-2.66; *P* < .001), Asian race, pregnancy at baseline, and marijuana use were associated with higher odds of reporting adverse effects. Use of inhaled corticosteroids did not appear to be associated with fewer adverse effects in participants with asthma (eTable 2 in the Supplement).

**Table 2.** Factors Associated With Any Adverse Effects After COVID-19 Vaccination

Characteristic^a^	OR (95% CI)	*P* value
Vaccine dose^b^	3.10 (2.89-3.34)	<.001
Age (per 10 y)	0.74 (0.72-0.76)	<.001
Brand		
BNT162b2	1 [Reference]	NA
mRNA-1273	2.00 (1.86-2.15)	<.001
JNJ-78436735	0.64 (0.52-0.79)	<.001
Female sex (vs all others)	1.65 (1.53-1.78)	<.001
Self-reported COVID-19 before vaccine	2.17 (1.77-2.66)	<.001
Race		
White	1 [Reference]	NA
Asian	1.49 (1.28-1.73)	<001
Black or African American	0.75 (0.58-0.97)	.03
Multiracial	1.18 (0.98-1.43)	.08
Other	1.07 (0.82-1.38)	.63
Asthma	0.77 (0.68-0.87)	<.001
Marijuana use	1.07 (1.03-1.11)	<.001
Anemia	0.83 (0.76-0.93)	.001
Pregnant at baseline	1.54 (1.15-2.06)	.004
Subjective social status (per unit increase)	0.97 (0.95-0.99)	.009
Sleep apnea	0.94 (0.86-1.04)	.23
Diabetes	0.92 (0.80-1.06)	.24
Influenza shot last year	0.95 (0.85-1.06)	.33
HIV	0.86 (0.63-1.17)	.33
Heart failure	1.13 (0.87-1.48)	.36
Tobacco use	1.02 (0.98-1.06)	.38
Hispanic ethnicity	0.94 (0.81-1.09)	.40
Myocardial infarction	0.93 (0.79-1.10)	.41
COPD	1.09 (0.89-1.32)	.41
Hypertension	1.03 (0.95-1.12)	.45
Stroke or TIA	1.08 (0.89-1.32)	.48
Coronary heart disease	0.93 (0.72-1.19)	.55
Atrial fibrillation	0.99 (0.87-1.14)	.94
Immunodeficiency	1.00 (0.85-1.17)	.99

Abbreviations: COPD, chronic obstructive pulmonary disease; NA, not applicable; OR, odds ratio; TIA, Transient Ischemic Attack.

^a^ All factors were entered into the multivariable logistic regression model simultaneously.

^b^ Adverse effects after 2 doses of BNT162b2 or mRNA-1273 or 1 dose of JNJ-78436735 compared with reference of adverse effects after 1 dose of BNT162b2 or mRNA-1273.

In a multivariable logistic regression model for the outcome of severe or very severe adverse effects (compared with no adverse effects, very mild, mild, or moderate), the strongest factor associated with severe or very severe adverse effects was vaccine dose (Table 3). Older age, receiving a influenza shot last year, asthma, obstructive sleep apnea, and higher subjective social status were associated with lower odds of reporting severe or very severe adverse effects. Receipt of mRNA-1273 vaccine (vs BNT162b2), female sex, and prior COVID-19 were associated with higher odds of reporting severe or very severe adverse effects.

**Table 3.** Factors Associated With Severe or Very Severe Adverse Effects After COVID-19 Vaccination

Characteristic^a^	OR (95%CI)	*P* value
Vaccine dose^b^	2.59 (2.20-3.06)	<.001
Brand		
BNT162b2	1 [Reference]	NA
mRNA-1273	1.88 (1.63-2.17)	<.001
JNJ-78436735	1.03 (0.67-1.58)	.89
Self-reported COVID-19 before vaccine	2.10 (1.63-2.70)	<.001
Female sex (vs all others)	1.68 (1.39-2.02)	<.001
Age (per 10 y)	0.89 (0.85-0.94)	<.001
Influenza shot last year	0.71 (0.58-0.87)	<.001
Asthma	0.77 (0.62-0.94)	.01
Sleep apnea	0.78 (0.63-0.95)	.01
Subjective social status (per unit increase)	0.95 (0.91-0.99)	.02
Race		
White	1 [Reference]	NA
Asian	1.23 (0.94-1.61)	.14
Black or African American	1.11 (0.65-1.91)	.71
Multiracial	1.07 (0.76-1.49)	.70
Other	1.25 (0.79-1.98)	.33
Immunodeficiency	0.75 (0.54-1.03)	.08
Tobacco use	1.07 (0.99-1.16)	.09
Heart failure	0.62 (0.34-1.11)	.11
Hispanic ethnicity	1.22 (0.94-1.59)	.14
Pregnant at baseline	1.40 (0.76-2.59)	.28
Hypertension	0.91 (0.76-1.09)	.29
Atrial fibrillation	0.86 (0.62-1.20)	.38
Diabetes	1.09 (0.80-1.49)	.59
Anemia	0.96 (0.77-1.18)	.67
Stroke or TIA	0.91 (0.58-1.43)	.68
COPD	0.93 (0.61-1.42)	.73
Marijuana use	0.99 (0.92-1.07)	.83
Coronary heart disease	0.95 (0.52-1.74)	.87
HIV	0.97 (0.41-2.30)	.95
Myocardial infarction	1.00 (0.67-1.48)	.99

Abbreviations: COPD, chronic obstructive pulmonary disease; NA, not applicable; OR, odds ratio; TIA, Transient Ischemic Attack.

^a^ All factors were entered into the multivariable logistic regression model simultaneously.

^b^ Adverse effects after 2 doses of BNT162b2 or mRNA-1273 or 1 dose of JNJ-78436735 compared with reference of adverse effects after 1 dose of BNT162b2 or mRNA-1273.

Adverse effects and adverse effect severity varied across vaccine brands. Compared with participants receiving BNT162b2 vaccine, participants receiving mRNA-1273 had double the odds of reporting adverse effects (odds ratio, 2.00; 95% CI, 1.86 to 2.15; *P* < .001). Participants receiving JNJ-78436735 had lower odds of adverse effects compared with BNT162b2 (OR, 0.64; 95% CI, 0.52-0.79; *P* < .001). Compared with participants receiving BNT162b2 vaccine, participants receiving mRNA-1273 had 1.88 times (95% CI, 1.63-2.17) the odds of reporting severe or very severe adverse effects (*P* < .001). There was not a statistically significant difference in odds of severe or very severe adverse effects reported by participants receiving the JNJ-78436735 vaccine compared with the BNT162b2 vaccine (OR, 1.03; 95% CI, 0.67-1.58; *P* = .89).

## Discussion

In this real-world digital cohort of 19 586 people who reported receiving COVID-19 vaccination, serious adverse effects, such as anaphylaxis or allergy, were rare. Adverse effects were more common after the full vaccination dose, the mRNA-1273 vaccine, and in participants with younger age, female sex, prior COVID-19, Asian race, pregnancy at baseline, and marijuana use. Older age, Black or African American race, higher subjective social status, asthma, and anemia were associated with lower odds of reporting adverse effects.

The finding of low rates of serious adverse effects is consistent with data from randomized clinical trials and government-sponsored surveillance of vaccine safety.^5,6,7,8,9,10,11,12^ US government-sponsored surveillance has reported incidence of anaphylaxis of 4.5 to 5.1 cases per million doses administered, although this may be underreported if individuals did not seek care or received care at another facility.^10,13^ Other reports suggest patient-reported anaphylaxis or severe allergy may be more common, at 2%.^22^ Our study finding of participant-reported anaphylaxis or allergy in 0.3% is higher than surveillance reports but lower than reports of patient-reported adverse effects. Similarly, the array of adverse effects and finding of more adverse effects in younger participants in our study are consistent with adverse effects that were observed in randomized clinical trials and government-sponsored surveillance and that are listed on government-sponsored websites.^5,6,7,8,9,10,11,12,23^ Additionally, the array of adverse effects is similar to those identified through systematic reviews and meta-analysis of randomized trials and US-government-sponsored reporting.^24,25^

Because randomized clinical trials of vaccine safety and efficacy were conducted with single brands of vaccines, it is difficult to make comparisons regarding rates of adverse effects from different brands of vaccines and different studies. US government–sponsored surveillance reported rates of adverse effects with BNT162b2 and mRNA-1273 vaccines.^10^ Although it appeared that there were more adverse effects with mRNA-1273 than BNT162b2, the reports did not make formal comparisons between vaccine brands.^10^ This study enables comparisons across vaccine brands because it included participants who received any brand of vaccine, comes from a nongovernment source, and administered the same survey to all participants.

In this study, people with prior COVID-19 had greater odds of adverse effects and more severe adverse effects after COVID-19 vaccination. Data from randomized clinical trials were not conclusive with regard to the association between prior COVID-19 and adverse effects after vaccination.^5^ Other smaller real-world reports have also reported increased adverse effects in people with prior COVID-19.^22^ In this study, which included 895 participants with prior COVID-19, there was a strong association between prior COVID-19 and vaccine adverse effects.

To our knowledge, this is the first report of lower risk of vaccine adverse effects or severe adverse effects in individuals with asthma. A meta-analysis found that people with asthma may appear to have lower risk of COVID-19 than the general population.^26^ It is possible that the airway effects of asthma or immune effects of inhaled corticosteroids may affect the response to COVID-19 or COVID-19 vaccine. Rare adverse effects, such as thrombocytopenia, were reported by participants; rare reports of thrombocytopenia have also emerged in the literature.^27^

Racial differences in the risk of adverse effects were observed in our study, with people identifying as Asian more likely to report adverse effects and people identifying as Black or African American less likely to report adverse effects. However, we are unable to determine whether these differences are present because of differential reporting, different experience of receiving the vaccine, differences in other social determinants of health or experiences of health, differences in immune system reaction to vaccines, or other incompletely measured confounders.

This study provides important data for the public about adverse effects and vaccine safety that confirm data from randomized clinical trials and government-sponsored surveillance. Indeed, large digital cohort studies may provide a mechanism for simple, inexpensive, and independent postmarket surveillance for adverse effects of new drugs and devices. Additionally, this study found that groups including older individuals, males, people who identify as Black or African American, and people with asthma were less likely to experience adverse effects or severe adverse effects. The overall low rates of serious adverse effects and greater knowledge about patterns and factors associated with adverse effects may enhance public vaccine confidence and promote greater adoption of vaccination to enable global recovery from the pandemic. However, given the limited representativeness of this study, future studies should conduct targeted efforts to recruit representative populations. Previously, we have shown that recruiting through community organizations can promote participation in digital clinical studies.^28^

### Limitations

This study has limitations. Although the digital cohort study did include people from diverse groups, some groups, such as men, older adults, people belonging to minoritized racial and ethnic groups, rural residents, people reporting lower subjective social status, and non-US residents, are underrepresented, which may limit generalizability to all groups or populations outside of the United States. Given the online nature of the study, not all participants responded to all surveys. This may contribute to both measurement bias through undermeasurement of vaccine receipt or COVID-19 diagnosis and selection bias if participants from different groups or participants with adverse effects responded differentially to surveys. In previous studies, we found that self-reported COVID-19 test results appeared to accurately reflect COVID-19 diagnosis.^16^ Additionally, administration of surveys on a monthly basis could lead to measurement bias through inaccurate reporting of vaccine-related adverse effects. Because this study included data until May 19, 2021, this reflects the early experience with vaccination, and results could differ in later time periods or with other vaccines.

## Conclusions

In this real-world cohort, serious COVID-19 vaccine adverse effects were rare, and overall adverse effects were similar to industry and government reports. This independent evaluation enabled the comparison of adverse effects between vaccine manufacturers, noting that adverse effects were more common with mRNA-1273 compared with BNT162b2. Large digital cohort studies may provide a mechanism for independent postmarket surveillance of drugs and devices.

## Notes

Supplement. **eTable 1.** Characteristics of COVID-19 Citizen Science Study Participants **eTable 2.** Adverse Effects in Participants With Asthma by Inhaled Corticosteroid Use **eFigure.** Free-Text Entries for Other Vaccine Adverse Effects
