# Supplementary material for: Analysis of COVID-19 Vaccine Type and Adverse Effects Following Vaccination
Source: JAMA Netw Open. 2021 Dec 22;4(12):e2140364. doi: 10.1001/jamanetworkopen.2021.40364 (PMC8696570; doi:10.1001/jamanetworkopen.2021.40364)
Supplement: Supplement. — eTable 1. Characteristics of COVID-19 Citizen Science Study Participants eTable 2. Adverse Effects in Participants With Asthma by Inhaled Corticosteroid Use eFigure. Free-Text Entries for Other Vaccine Adverse Effects [file jamanetwopen-e2140364-s001.pdf]

Supplemental Online Content

Beatty AL, Peyser ND, Butcher XE, et al. Analysis of COVID-19 vaccine type and adverse effects following vaccination. *JAMA Netw Open.* 2021;4(12):e2140364. doi:10.1001/jamanetworkopen.2021.40364

eTable 1. Characteristics of COVID-19 Citizen Science Study Participants

eTable 2. Adverse Effects in Participants With Asthma by Inhaled Corticosteroid Use

eFigure. Free-Text Entries for Other Vaccine Adverse Effects

This supplemental material has been provided by the authors to give readers additional information about their work.

© 2021 Beatty AL et al. *JAMA Network Open.*

eTable 1. Characteristics of COVID-19 Citizen Science Study Participants

	All participants N = 65921 N (%)	Participants active 1/2021-5/2021 N = 46204 N (%)
US resident	62743 (95.2%)	42827 (97.5%)
Age, median (IQR)	43.0 (34.0, 57.0)	46.0 (34.0, 60.0)
Female sex assigned at birth	44027 (68.0%)	30255 (69.4%)
Gender identity		
Male	20452 (31.6%)	13140 (30.2%)
Female	43391 (67.0%)	29808 (68.4%)
Transgender Woman	89 (0.1%)	62 (0.1%)
Transgender Man	142 (0.2%)	101 (0.2%)
Genderqueer	510 (0.8%)	354 (0.8%)
Other	216 (0.3%)	159 (0.4%)
Race/Ethnicity		
White	56643 (88.4%)	38325 (88.7%)
Black or African American	1594 (2.5%)	1120 (2.6%)
Asian	5319 (8.3%)	3460 (8.0%)
Native Hawaiian or Pacific Islander	248 (0.4%)	189 (0.4%)
American Indian or Alaska Native	984 (1.5%)	724 (1.7%)
Other/Don't know	2539 (4.0%)	1709 (4.0%)
Hispanic ethnicity (yes: PR, Mexican, Other, Cuban)	5512 (8.5%)	3867 (8.9%)
Rural ZIP code	4248 (6.8%)	3013 (7.1%)
Lives in ZIP in lowest quintile for median household income	2557 (4.1%)	1656 (3.9%)
Subjective Social Status[a], mean ± SD	6.8 ± 1.7	6.8 ± 1.7
Highest Educational Level		
No high school degree	312 (0.5%)	138 (0.3%)
High school graduate (or equivalent)	2639 (4.1%)	1481 (3.4%)
College degree (including associate's)	34115 (52.7%)	22879 (52.5%)
Graduate degree	26866 (41.5%)	18568 (42.6%)
Other	827 (1.3%)	492 (1.1%)

© 2021 Beatty AL et al. *JAMA Network Open.*

	All participants<br>N = 65921<br>N (%)	Participants active 1/2021-5/2021<br>N = 46204<br>N (%)
Primary Employment		
Healthcare	13651 (20.7%)	8943 (20.4%)
Education	8018 (12.2%)	5225 (11.9%)
Retail	1817 (2.8%)	967 (2.2%)
Transportation	930 (1.4%)	555 (1.3%)
Arts, entertainment, and recreation	2012 (3.1%)	1264 (2.9%)
Hospitality and food services	1415 (2.1%)	894 (2.0%)
Finance and insurance	3506 (5.3%)	2340 (5.3%)
Scientific and technical services	7022 (10.7%)	4308 (9.8%)
Utilities	552 (0.8%)	313 (0.7%)
Construction	985 (1.5%)	621 (1.4%)
Manufacturing	1373 (2.1%)	930 (2.1%)
Other	24637 (37.4%)	17553 (40.0%)
Working from home		
100%	10159 (50.7%)	6991 (49.8%)
>=50%	3274 (16.3%)	2344 (16.7%)
<50%	2256 (11.3%)	1643 (11.7%)
None	4362 (21.8%)	3055 (21.8%)
Regular contact with 65+	25083 (38.1%)	17974 (40.9%)
Flu shot in the past year	50893 (77.2%)	35723 (81.4%)
Body Mass Index, mean ± SD	27.5 ± 6.7	27.5 ± 6.7
Medical Condition		
Hypertension	13244 (21.0%)	9556 (22.8%)
Diabetes	2952 (4.7%)	2152 (5.1%)
Coronary Artery Disease	1823 (2.9%)	1342 (3.2%)
Myocardial Infarction	739 (1.2%)	541 (1.3%)
Congestive heart failure	518 (0.8%)	386 (0.9%)
Stroke or TIA	984 (1.6%)	735 (1.7%)
Atrial fibrillation	1961 (3.1%)	1500 (3.6%)
Sleep apnea	6650 (10.6%)	4714 (11.2%)
COPD	1204 (1.9%)	852 (2.0%)
Asthma	6447 (10.2%)	4249 (10.1%)

© 2021 Beatty AL et al. *JAMA Network Open.*

	All participants<br>N = 65921<br>N (%)	Participants active 1/2021-5/2021<br>N = 46204<br>N (%)
Immunodeficiency	1651 (2.6%)	1199 (2.9%)
HIV	381 (0.6%)	284 (0.7%)
Anemia	6686 (10.6%)	4634 (11.0%)
Pregnant (at baseline)	801 (1.3%)	576 (1.4%)
Current smoking	3303 (5.4%)	1815 (4.4%)
Current marijuana use	6335 (10.4%)	4045 (9.9%)
COVID-19 (at baseline or incident before date of vaccine)	3018 (4.6%)	2575 (5.9%)

[a] Subjective social status is rated 1-10, with 10 representing the highest perceived social status.
Abbreviations: COPD = chronic obstructive pulmonary disease; HIV = human immunodeficiency virus; IQR = interquartile range; SD = standard deviation; TIA = transient ischemic attack

© 2021 Beatty AL et al. *JAMA Network Open.*

eTable 2. Adverse Effects in Participants With Asthma by Inhaled Corticosteroid Use

	All with asthma	Asthma + medication list includes inhaled corticosteroids	Asthma + medication list does not include inhaled corticosteroids	p value
N with at least one dose	1950	387	1563	
Adverse effects				
N with reporting adverse effects	1949	387	1562	
Fever	366 (18.8%)	81 (20.9%)	285 (18.2%)	0.23
Chills	503 (25.8%)	99 (25.6%)	404 (25.9%)	0.91
Fatigue	1066 (54.7%)	224 (57.9%)	842 (53.9%)	0.16
Sore/scratchy throat	113 (5.8%)	17 (4.4%)	96 (6.1%)	0.19
Muscle pain	911 (46.7%)	177 (45.7%)	734 (47.0%)	0.66
Joint pain	431 (22.1%)	89 (23.0%)	342 (21.9%)	0.64
Headache	729 (37.4%)	139 (35.9%)	590 (37.8%)	0.50
Other pain	116 (6.0%)	23 (5.9%)	93 (6.0%)	0.99
Redness/swelling at the injection site	419 (21.5%)	108 (27.9%)	311 (19.9%)	<.001
Rash other than at the injection site	45 (2.3%)	10 (2.6%)	35 (2.2%)	0.69
Allergic reaction/anaphylaxis	7 (0.4%)	1 (0.3%)	6 (0.4%)	0.71
Other	193 (9.9%)	39 (10.1%)	154 (9.9%)	0.90
None of the above	404 (20.7%)	65 (16.8%)	339 (21.7%)	0.03
Median duration of adverse effects, (IQR)	2 (1, 3)	2 (1, 3)	2 (1, 3)	0.91
Adverse effect severity				
N with reporting adverse effect severity	1717	363	1354	
Very Mild	270 (15.7%)	59 (16.3%)	211 (15.6%)	0.76
Mild	580 (33.8%)	125 (34.4%)	455 (33.6%)	0.77
Moderate	570 (33.2%)	119 (32.8%)	451 (33.3%)	0.85
Severe	119 (6.9%)	19 (5.2%)	100 (7.4%)	0.15
Very Severe	7 (0.4%)	0	7 (0.5%)	0.17

Abbreviations: IQR- Interquartile range

© 2021 Beatty AL et al. *JAMA Network Open.*

eFigure 1. Free-Text Entries for Other Vaccine Adverse Effects

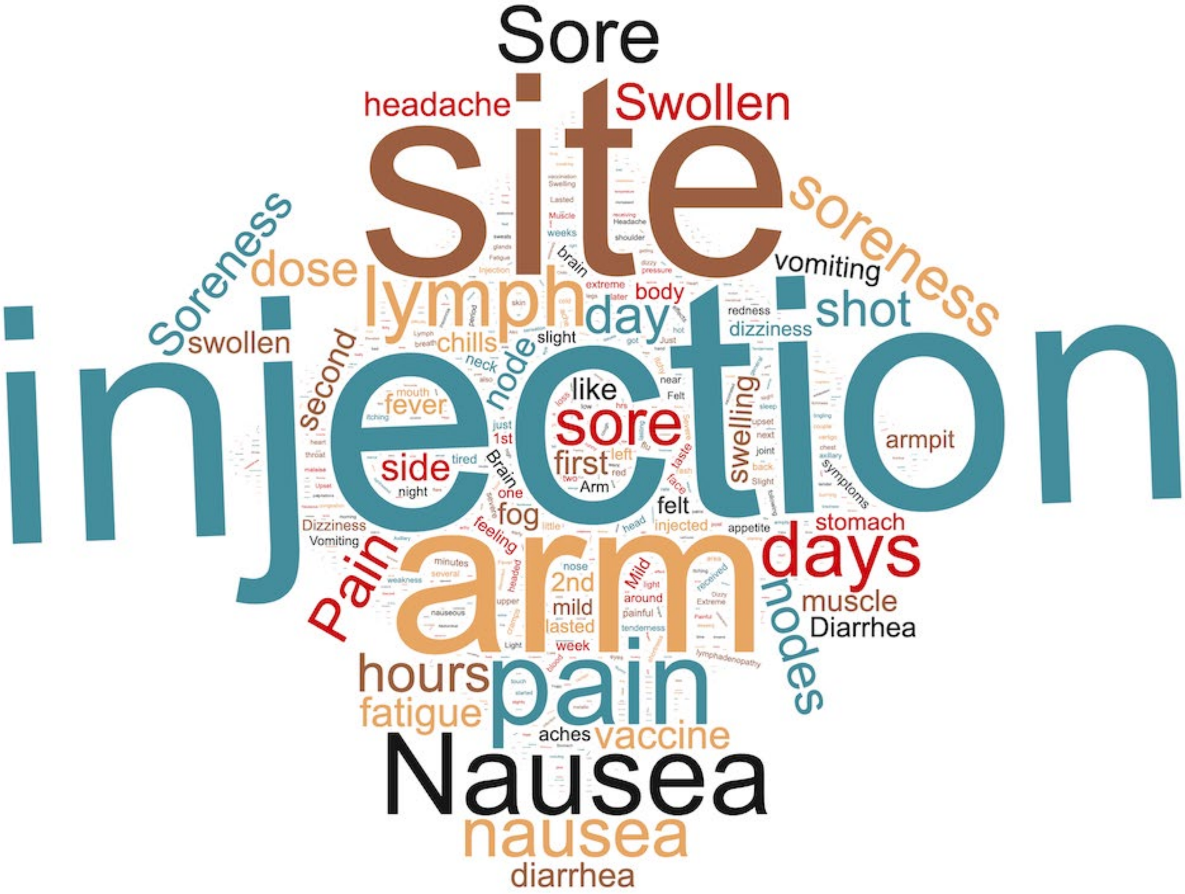

© 2021 Beatty AL et al. *JAMA Network Open.*
